# Effective Detection and Monitoring of Glioma Using [^18^F]FPIA PET Imaging

**DOI:** 10.3390/biomedicines9070811

**Published:** 2021-07-13

**Authors:** Vessela Vassileva, Marta Braga, Chris Barnes, Justyna Przystal, Ali Ashek, Louis Allott, Diana Brickute, Joel Abrahams, Keittisak Suwan, Angel M. Carcaboso, Amin Hajitou, Eric O. Aboagye

**Affiliations:** 1 Department of Surgery and Cancer, Division of Cancer, Imperial College London, Hammersmith Campus, Du Cane Road, London W12 0NN, UK; m.braga13@imperial.ac.uk (M.B.); chris.barnes@imperial.ac.uk (C.B.); l.allott@imperial.ac.uk (L.A.); d.brickute@imperial.ac.uk (D.B.); joel.abrahams@imperial.ac.uk (J.A.); 2 Department of Medicine, Division of Brain Sciences, Imperial College London, Hammersmith Campus, Burlington Danes, London W12 0NN, UK; justyna.przystal@kispi.uzh.ch (J.P.); keittisak.suwan@imperial.ac.uk (K.S.); a.hajitou@imperial.ac.uk (A.H.); 3 Department of Medicine, Faculty of Medicine, Imperial College London, Hammersmith Campus, Du Cane Road, London W12 0NN, UK; m.ashek@imperial.ac.uk; 4 Institute de Recerca Sant Joan de Deu, 08950 Barcelona, Spain; amontero@fsjd.org

**Keywords:** [^18^F]FPIA PET imaging, fatty acid metabolism, proliferation, glioma

## Abstract

Background: Reprogrammed cellular metabolism is a cancer hallmark. In addition to increased glycolysis, the oxidation of acetate in the citric acid cycle is another common metabolic phenotype. We have recently developed a novel fluorine-18-labelled trimethylacetate-based radiotracer, [^18^F]fluoro-pivalic acid ([^18^F]FPIA), for imaging the transcellular flux of short-chain fatty acids, and investigated whether this radiotracer can be used for the detection of glioma growth. Methods: We evaluated the potential of [^18^F]FPIA PET to monitor tumor growth in orthotopic patient-derived (HSJD-GBM-001) and cell line-derived (U87, LN229) glioma xenografts, and also included [^18^F]FDG PET for comparison. We assessed proliferation (Ki-67) and the expression of lipid metabolism and transport proteins (CPT1, SLC22A2, SLC22A5, SLC25A20) by immunohistochemistry, along with etomoxir treatment to provide insights into [^18^F]FPIA uptake. Results: Longitudinal PET imaging showed gradual increase in [^18^F]FPIA uptake in orthotopic glioma models with disease progression (*p* < 0.0001), and high tumor-to-brain contrast compared to [^18^F]FDG (*p* < 0.0001). [^18^F]FPIA uptake correlated positively with Ki-67 (*p* < 0.01), SLC22A5 (*p* < 0.001) and SLC25A20 (*p* = 0.001), and negatively with CPT1 (*p* < 0.01) and SLC22A2 (*p* < 0.01). Etomoxir reduced [^18^F]FPIA uptake, which correlated with decreased Ki-67 (*p* < 0.05). Conclusions: Our findings support the use of [^18^F]FPIA PET for the detection and longitudinal monitoring of glioma, showing a positive correlation with tumor proliferation, and suggest transcellular flux-mediated radiotracer uptake.

## 1. Introduction

Glioma is the most common primary malignant brain tumor in adults, and approximately 55% of patients are diagnosed with highly-aggressive disease, known as grade IV astrocytoma, glioblastoma multiforme, or glioblastoma [1,2,3]. Unfortunately, the prognosis and quality of life of these patients is dismal, with median survival of about a year, despite treatment, which may include surgical resection, radiotherapy, and chemotherapy or a combination of these [1,2].

While MRI and CT are the gold-standard for diagnosis of central nervous system tumors, precise delineation of tumor volume during planning of local treatments, such as surgical resection or radiotherapy is challenging [4]. Furthermore, both treatment and tumor recurrence can induce similar local tissue changes resulting in the impairment of the blood–brain barrier, which are difficult to distinguish with current imaging protocols [5]. For instance, T1-weighted MR images obtained months after a baseline scan can be confounded by pseudoprogression [5,6]. Therefore, the development of imaging techniques that reliably differentiate tumor foci could help guide treatment planning, monitor tumor growth and response to treatment, and facilitate the development of novel therapies.

Several studies have demonstrated that glioma cells primarily use fatty acid metabolism for energy production and proliferation [7,8,9]. Fatty acids have critical roles in energy storage, membrane synthesis, and the generation of signaling molecules, including cholesterol and other steroid hormones, driving signaling through the mevalonate pathway, which is highly active in glioma [10,11]. Incidentally, short-chain fatty acids are rapidly taken up by tumors. For example, models of malignant glioma, hepatocellular carcinoma, and breast and prostate cancer show that acetate contributes to most of the oxidative activity for energy production to support tumor growth [12,13,14,15]. In glioma, glucose has been reported to contribute to less than 50% of the carbons to the acetyl-CoA pool, with acetate, and not glutamine, representing a unique substrate for oxidation in the citric acid cycle [12]. Thus, this distinct metabolic phenotype is likely an adaptation to sustain the high biosynthetic and bioenergetic demands of malignant tumor growth. While [^11^C]acetate has been exploited for one of its several fates—synthesis of fatty acids—in other cancers [16], its role in brain tumors has recently taken center-stage [12]. To that end, we have designed a novel radiotracer, based on trimethylacetate (2,2-dimethylpropionic acid, pivalic acid), [^18^F]fluouro-pivalic acid ([^18^F]FPIA), for imaging transcellular flux of short-chain fatty acids into tumor cells [17,18]. Pivalic acid is converted to pivaloyl-CoA by acyl-CoA synthases; however, unlike the CoA-thioesters of acetate or isobutyrate, pivaloyl-CoA cannot be oxidized to carbon dioxide in mammalian cells [19,20]. Consequently, pivaloyl-CoA accumulates in cells, becoming a substrate for acyl-CoA transferases, generating pivaloyl conjugates, including pivaloyl-carnitine. The formation and urinary excretion of pivaloyl-carnitine is the dominant route of elimination of pivalic acid in humans [19,20]. We have previously shown that [^18^F]FPIA, but not fluoroacetate, can modulate carnitine and short-/long-chain fatty acyl-carnitine levels in cells, with rapid radiotracer accumulation in subcutaneously implanted brain, breast and prostate cancer xenografts, and lower brain uptake compared to [^18^F]fluorodeoxyglucose ([^18^F]FDG) [18]. We, therefore, hypothesized that [^18^F]FPIA PET could be employed in the reliable detection of brain tumors.

To provide further insights into the potential use of this radiotracer in glioma, the current study investigated whether [^18^F]FPIA PET permits the monitoring of tumor growth in orthotopic glioma models, including patient-derived and tumor cell-derived xenografts, and also examined markers of proliferation, and lipid metabolism and transport, along with etomoxir treatment in relation to radiotracer uptake.

## 2. Materials and Methods

### 2.1. Radiotracer Synthesis

Radiosynthesis of [^18^F]FPIA was performed as previously described [8,9], with end-of-synthesis radiochemical yield of 19.4 ± 1.9% and purity of >99%. [^18^F]FDG was obtained from PETNET Solutions (Middlesex, UK).

### 2.2. Cell Culture

Primary human glioma cells, HSJD-GBM-001, were established at the Hospital Sant Joan de Deu, Barcelona, Spain, and grown in differentiation culture tumor stem medium (TSM) composed of 50% Neurobasal-A Medium (1×), 50% DMEM/F12 (1×), 1% HEPES buffer solution (1 M), 1% sodium pyruvate MEM (100 mM), 1% MEM Non-Essential Amino Acids solution 10 mM (100×), 1% GlutaMAX supplement, 1% Antibiotic-Antimycotic (100×), and 10% FBS.

Human glioma U87 and LN229 cell lines (American Type Culture Collection) were cultured in DMEM. Media were supplemented with 2 mM l-glutamine and 10% FBS, and cells were maintained at 37 °C in a humidified atmosphere containing 5% CO_2_. All experiments were conducted within 6 months of cell line purchase and/or authentication.

### 2.3. Tumor Models

All animal experiments were performed in accordance with the United Kingdom Home Office Guidance on the Operation of the Animals (Scientific Procedures) Act 1986 Amendment regulations 2012 and National Cancer Research Institutes Guidelines for the welfare and use of animals in cancer research [21]. Studies were conducted under Project License Number PPL 70/8651. Tumor xenografts were established in female Balb/C nude mice (6–8 weeks old, Charles River).

Orthotopic tumor models were generated using patient-derived HSJD-GBM-001, U87 and LN229 glioma cell lines. HSJD-GBM-001 cells were grown as tumor spheres in TSM supplemented with B27 (1X), human FGF and EGF (20 ng/mL, each), PDGF-AA and PDGF-BB (10 ng/mL, each), and heparin (2 µg/mL). Prior to intracranial injection, the tumor sphere cell suspension was strained (70 μm cell strainer) and kept on ice in base TSM. The U87 and LN229 cells were cultured as described above.

Mice were anesthetized with isoflurane, and 1cm sagittal incision was made under sterile conditions. Tumor cell injection coordinates were established in a stereotactic frame: 2 mm to the right of the bregma, which served as the origin, and 1mm to the coronal suture. The skull was drilled (3.5 mm deep) and tumor cells (5 × 10^5^, 6 µL) were injected into the drilled cavity over 6 min, and the scalp then sutured. Mice were monitored post-operatively until they became ambulant and retained normal activity (~15 min recovery time).

For maximal tumor harvest for the biodistribution and molecular pathology assays, subcutaneous tumors were generated using HSJD-GBM-001 and U87 glioma cells. Briefly, 1 × 10^6^ tumor cells were injected subcutaneously into the neck region of mice and tumor growth was evaluated using caliper measurements, and calculated as tumor volume using the formula: volume = π/6 (length × width × height).

### 2.4. Imaging

Mice were anesthetized with isoflurane, and the lateral tail vein was cannulated for the i.v. administration of [^18^F]FPIA or [^18^F]FDG. Imaging was performed using the Inveon small-animal multimodality PET/CT system (Siemens Healthcare Molecular Imaging, Siemens Healthcare GmbH, Erlangen, Germany). Following CT scan, [^18^F]FPIA or [^18^F]FDG was administered (5MBq per mouse) and dynamic emission scans were acquired in list-mode format for 60 min using conventional full-ring whole-body PET.

Mice with orthotopic HSJD-GBM-001 tumors were imaged once per week over a period of 21 days for a total of three scans. Mice with orthotopic U87 and LN229 tumors were imaged twice in total, on days 22 and 29, and on days 27 and 70 post-tumor implantation, respectively, guided by bioluminescent imaging for tumor growth confirmation as previously described [22,23]. Mice with subcutaneous tumors (HSJD-GBM-001, U87) underwent single [^18^F]FPIA PET imaging.

PET image data were sorted into 0.5mm sinogram bins, and 33 time-frames and images were reconstructed using 2D-ordered subsets expectation maximization (2D-OSEM) algorithm with CT-based attenuation correction, with frame durations of 12 × 5 s, 4 × 15 s, 6 × 30 s, 11 × 300 s. The reconstructed CT and PET images were analyzed using the Inveon Research Workplace software (Siemens Healthcare Molecular Imaging, Siemens Healthcare GmbH, Erlangen, Germany).

Regions of interest were generated, and tumor time-activity curves (TACs) were calculated from PET images and co-registered with CT images. Decay corrected tumor TACs were generated and normalized to whole-body activity (MBq/mL) to obtain normalized uptake values (NUVs); NUVs at 40–60 min post-injection, when equilibrium was sustained, were compared.

### 2.5. [^18^F]FPIA Biodistribution

The biodistribution of [^18^F]FPIA was evaluated in the same mice that underwent PET imaging (subcutaneous tumors only, due to the challenges in accurately excising orthotopic tumors from normal brain tissue). Briefly, immediately after PET scanning, mice were sacrificed by exsanguination via cardiac puncture and select tissues were collected for radioactivity measurement using γ-counting (Wizard 2480 Automatic Gamma Counter, Perkin Elmer, Beaconsfield, UK). Radiotracer biodistribution was calculated as percentage of injected dose per gram of tissue (%injected activity/g) and normalized to blood. Tumor tissues were then processed for immunohistochemistry.

### 2.6. Effect of Etomoxir on [^18^F]FPIA Tumor Uptake

We investigated whether acute pharmacological inhibition of fatty acid oxidation and mitochondrial function, using etomoxir [24], can influence [^18^F]FPIA tumor uptake. We used subcutaneous patient-derived (HSJD-GBM-001) and tumor-cell derived (U87) xenografts to assess [^18^F]FPIA uptake by PET imaging and ex vivo γ-counting, 24 h post-treatment [25]. Briefly, mice with similar tumor volumes were allocated to two groups: (1) control (PBS, 100 µL, i.p.) and (2) etomoxir (20 mg/kg, 100 µL, i.p.), *n* = 5 mice per group; 24 h after PBS or etomoxir administration, mice underwent [^18^F]FPIA PET/CT imaging as described above. Mice were then sacrificed (upon imaging completion, 60 min post [^18^F]FPIA injection) and tumor tissues were collected for γ-counting and immunohistochemistry.

### 2.7. Immunohistochemistry

Formalin-fixed, paraffin-embedded tumor tissues were sectioned for immunohistochemistry. We evaluated the expression of Ki-67, and proteins involved in lipid metabolism and transport, including carnitine-palmitoyl-transferase-1 (CPT1), solute carrier family 22 member 2 (SLC22A2) organic cation transporter, solute carrier family 22 member 5 (SLC22A5) organic cation transporter/sodium-dependent high affinity carnitine transporter, and solute carrier family 25 member 20 (SLC25A20) carnitine-acylcarnitine translocase. Briefly, after antigen retrieval, sections were incubated with primary antibodies (Ki-67, 1:1000; CPT1, 1:1000; SLC22A2, 1:250; SLC22A5, 1:500; SLS25A20, 1:250; Abcam) overnight, and then with relative biotin-conjugated secondary antibodies (Ventana Discovery DAB Map Kit; Roche), and counterstained with hematoxylin, and mounted with DPX (Sigma Aldrich). Image acquisition of tumor sections was performed using the AxioScan digital slide scanner (Zeiss) and image analyses with QuPath software (Queen’s University Belfast, Northern Ireland) software for quantification of positively-stained cells [26]. Briefly, regions of interest were defined to include viable tumor tissue, excluding necrotic areas. Cell-based analysis was performed with automated cell segmentation based on color: hematoxylin in the blue and DAB in the red channel. To avoid artefacts, a threshold for minimum cell area and variance of hematoxylin staining was set. Positive cells were selected on the basis of mean and maximum intensity of DAB. Data were generated by calculating the percentage of the DAB-reactive cells in relation to the total number of cells in the regions of interest.

### 2.8. Statistical Analyses

Data were plotted and analyzed using Prism, version 7.0 (GraphPad Software) and expressed as mean ± standard deviation. Differences between groups were assessed by unpaired Student’s and Multiple t tests, ANOVA, and Tukey’s multiple comparisons tests. Pearson linear regression analyses were performed to evaluate correlations between [^18^F]FPIA uptake (NUVs), proliferation indices, and lipid metabolism-associated proteins. Results were considered statistically significant at *p* < 0.05.

## 3. Results

### 3.1. [^18^F]FPIA Uptake Reflected Orthotopic Glioma Growth

**Figure 1:**
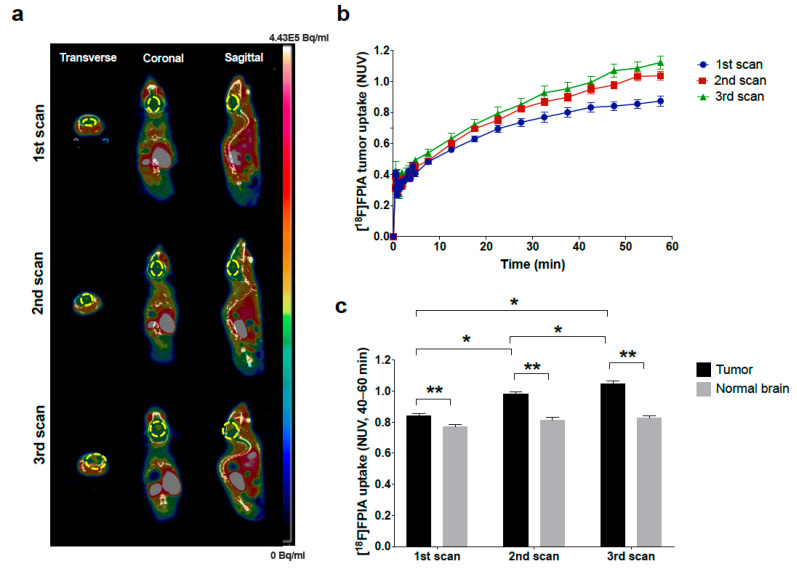
[^18^F]FPIA PET imaging of orthotopic patient-derived glioma xenograft growth. Mice with HSJD-GBM-001 tumors underwent weekly [^18^F]FPIA PET/CT imaging for a total of three scans. (**a**) Representative [^18^F]FPIA PET/CT images of consecutive scans. (**b**) Tumor time activity curves for [^18^F]FPIA (NUVs). (**c**) Tumor and normal brain uptake of [^18^F]FPIA (NUVs, 40–60 min). [^18^F]FPIA tumor uptake increased significantly with each scan (* *p* < 0.0001, 1st vs. 2nd; *p* < 0.05, 2nd vs. 3rd; *p* < 0.0001, 1st vs. 3rd; 2-way ANOVA). Significantly higher [^18^F]FPIA uptake was observed in tumor lesions compared to normal brain (** *p* < 0.05, 1st vs. 2nd; *p* < 0.0001, 2nd vs. 3rd and 1st vs. 3rd; 2-way ANOVA).

**Figure 2:**
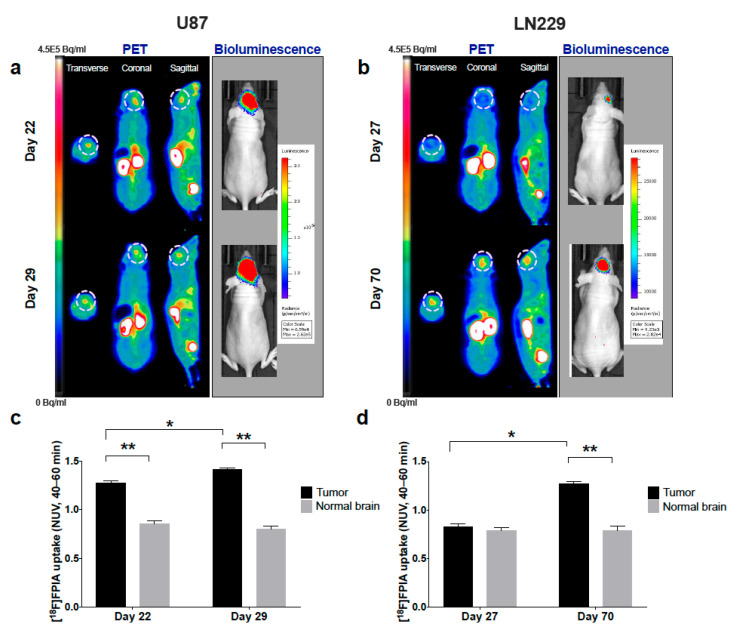
[^18^F]FPIA PET imaging of orthotopic glioma xenograft growth. Tumor models were generated using U87 and LN229 glioma cells, and mice underwent [^18^F]FPIA PET and bioluminescence imaging twice post-tumor cell implantation. Representative PET and bioluminescence images of (**a**) U87 and (**b**) LN229 tumors over time. Tumor and normal brain uptake of [^18^F]FPIA (NUVs, 40–60 min) in (**c**) U87 and (**d**) LN229 tumors. ^18^[F]FPIA tumor uptake significantly increased over time for U87 and LN229 tumors (* *p* = 0.001, *p* < 0.0001, respectively, 2-way ANOVA). Significantly higher [^18^F]FPIA uptake was observed in U87 tumor lesions compared to normal brain for both scans (** *p* < 0.0001, 2-way ANOVA), and at day 70 in LN229 tumors (** *p* < 0.0001, 2-way ANOVA).

Longitudinal PET imaging of mice with orthotopic patient-derived HSJD-GBM-001 tumors demonstrated significantly increased focal uptake of [^18^F]FPIA over time (1st < 2nd < 3rd scan, *p* < 0.0001, 2-way ANOVA), Figure 1. Of note, while 6/6 mice underwent the first and second scans, only 2/6 mice had the third scan, since 4/6 animals had aggressive disease progression and had to be humanely sacrificed before the third scan could be acquired (Figure 1a,b). [^18^F]FPIA uptake was significantly higher in tumor lesions compared to normal brain tissue for all three scans (*p* < 0.05, 1st scan, and *p* < 0.0001 for both 2nd and third scan, 2-way ANOVA), Figure 1c. [^18^F]FPIA uptake also increased over time in two additional orthotopic glioma models, U87 and LN229, reflecting tumor growth as confirmed by bioluminescence imaging (Figure 2a,b). Tumor-to-brain contrast was significantly high in both models, both on Day 22 and 29 for the faster growing U87 model (*p* < 0.0001, 2-way ANOVA), and only on Day 70 for the slower growing LN229 model (*p* < 0.0001, 2-way ANOVA), Figure 2c,d.

### 3.2. PET Imaging Showed Higher Tumor-to-Normal Brain Uptake of [^18^F]FPIA Compared with [^18^F]FDG

**Figure 3:**
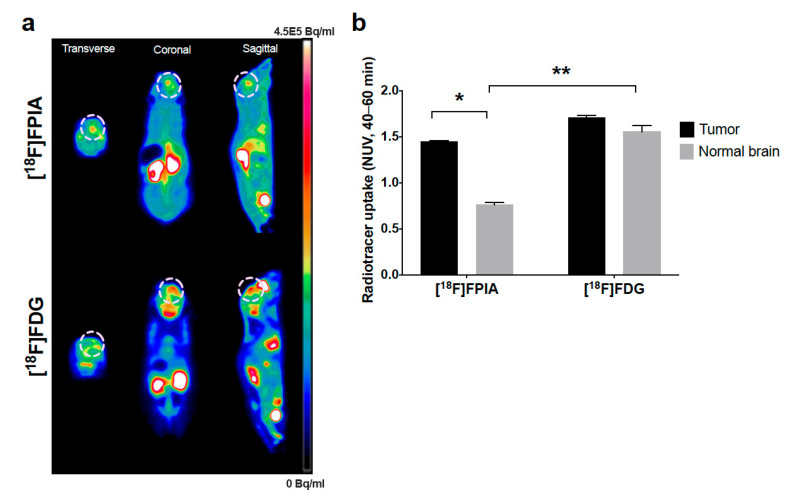
[^18^F]FPIA and [^18^F]FDG PET imaging of orthotopic U87 glioma model. (**a**) Representative [^18^F]FPIA and [^18^F]FDG PET images and (**b**) tumor and normal brain uptake of [^18^F]FPIA and [^18^F]FDG (NUVs, 40–60 min). Compared with normal brain, the uptake of [^18^F]FPIA was significantly higher in tumor lesions (* *p* < 0.0001, 2-way ANOVA), whereas, similar uptake was observed for [^18^F]FDG. Normal brain uptake of [^18^F]FDG was significantly higher than [^18^F]FPIA (** *p* < 0.0001, 2-way-ANOVA).

We performed [^18^F]FPIA and [^18^F]FDG PET imaging in mice with orthotopic U87 tumors to directly compare the uptake of each radiotracer in normal brain and tumor lesions (Figure 3a). [^18^F]FPIA uptake was 2-fold higher in tumor lesions compared to normal brain (*p* < 0.0001, 2-way ANOVA), whereas [^18^F]FDG uptake was similar in both normal and tumor tissue; normal brain uptake of [^18^F]FPIA was lower compared to [^18^F]FDG (*p* < 0.0001, 2-way ANOVA) Figure 3b.

### 3.3. Effect of Etomoxir on [^18^F]FPIA Tumor Uptake

**Figure 4:**
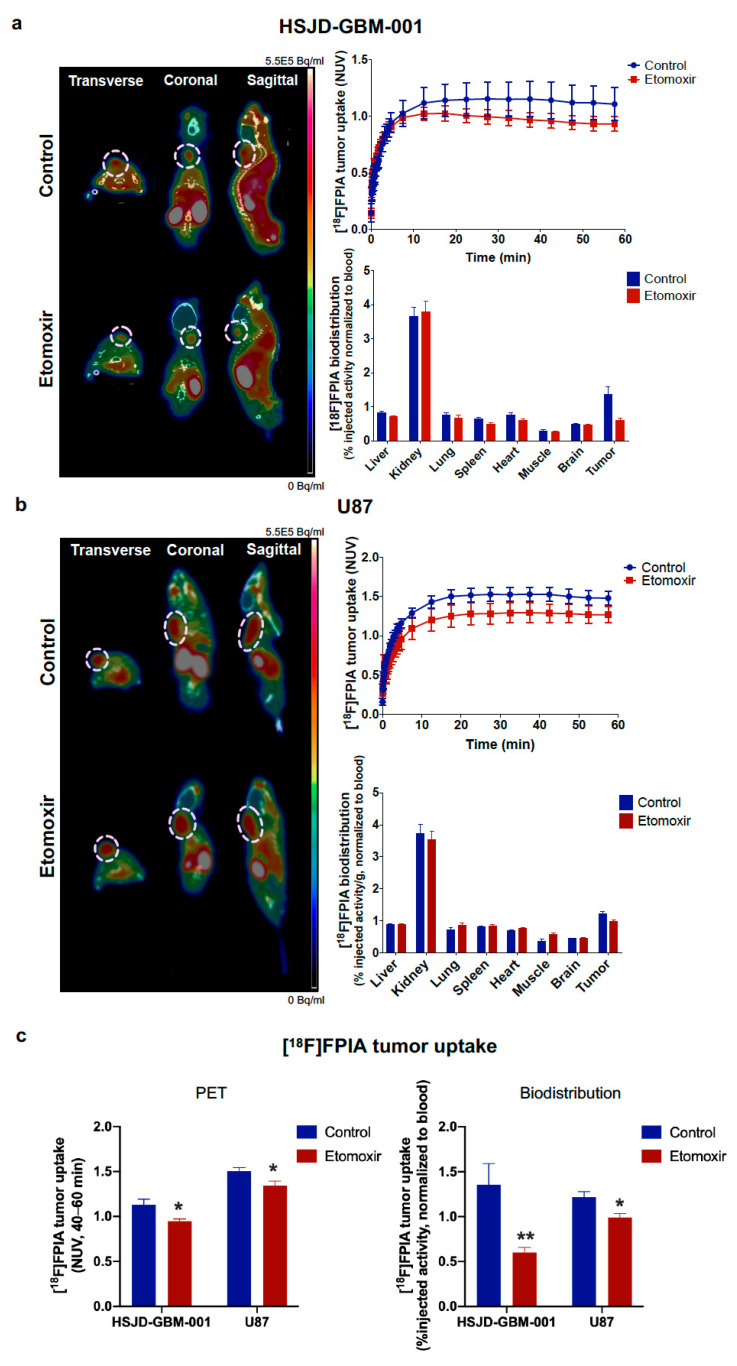
Effect of etomoxir on [^18^F]FPIA uptake in subcutaneous HSJD-GBM-001 and U87 glioma xenografts. [^18^F]FPIA uptake was evaluated in control and etomoxir-treated mice using PET imaging and ex vivo biodistribution studies. Representative PET images, tumor time activity curves (NUVs) and biodistribution (%injected radioactivity/g normalized to blood, ex-vivo γ-counting) of [^18^F]FPIA in (**a**) U87 and (**b**) HSJD-GBM-001 tumors, and (**c**) [^18^F]FPIA tumor uptake (NUVs, 40–60 min and %injected activity/g normalized to blood) in control and etomoxir-treated mice. Treatment with etomoxir significantly decreased [^18^F]FPIA uptake (NUVs, 40–60 min and % injected radioactivity/g, * *p* = 0.01 and ** *p* < 0.005, respectively, Student’s t tests) in both HSJD-GBM-001 and U87 tumors. No significant changes in radiotracer uptake were observed in the blood or any of the other tissues examined in response to etomoxir.

To examine whether [^18^F]FPIA uptake can be altered by disturbance of fatty acid metabolism, we used etomoxir to acutely disrupt fatty acid metabolism/mitochondrial function [24]. Radiotracer uptake was significantly decreased 24 h post-etomoxir treatment in both HSJD-GBM-001 and U87 tumors; no significant changes in radiotracer uptake were observed in response to etomoxir in the blood or any of the other examined tissues (Figure 4a,b); *p* = 0.01, PET NUVs 40–60 min for both xenografts, and *p* < 0.005 and *p* = 0.01, %injected radioactivity/g normalized to blood, respectively (Student’s *t* test) (Figure 4c).

### 3.4. [^18^F]FPIA Uptake Correlated with Tumor Proliferation and Expression of Lipid Metabolism-Associated Proteins

**Figure 5:**
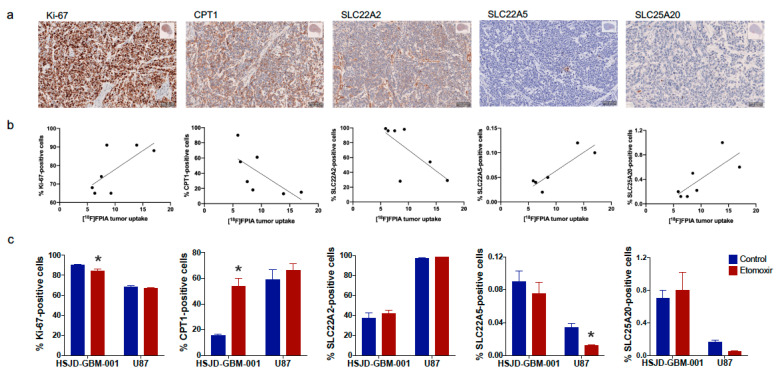
[^18^F]FPIA uptake in relation to tumor proliferation and lipid metabolism-associated proteins in subcutaneous HSJD-GBM-001 and U87 glioma xenografts. Tumor proliferation (Ki-67) and the expression of lipid metabolism-associated proteins (CPT1, SLC22A2, SLC22A5, SLS25A20) were assessed using immunohistochemistry. (**a**) Representative images of marker expression in HSJD-GBM-001 tumors (50 μm), (**b**) Correlation between [^18^F]FPIA tumor uptake and marker expression in both HSJD-GBM-001 and U87 tumors, (**c**) Tumor proliferation and lipid metabolism-associated protein expression in response to etomoxir treatment were assessed. Treatment with etomoxir significantly decreased Ki-67 and increased CPT1 expression in HSJD-GBM-001 tumors (* *p* < 0.05 and *p* = 0.0001, 2-way ANOVA, respectively), and decreased SLC22A5 expression in U87 tumors (* *p* < 0.05, 2-way ANOVA).

We assessed tumor proliferation (Ki-67) and the expression of proteins involved in lipid metabolism and transport (CPT1, SLC22A2, SLC22A5, SLS25A20) by immunohistochemistry. We correlated marker expression with [^18^F]FPIA uptake from the ex-vivo biodistribution data in subcutaneous HSJD-GBM-001 and U87 tumors. Representative images of protein expression are shown in Figure 5a. Linear regression analyses showed positive correlations with Ki-67 (R^2^ = 0.5, *p* < 0.01), SLC22A5 (R^2^ = 0.8, *p* < 0.001) and SLC25A20 (R^2^ = 0.6, *p* = 0.001), and negative correlations with CPT1 (R^2^ = 0.5, *p* < 0.01) and SLC22A2 (R^2^ = 0.5, *p* < 0.01), Table 1, Figure 5c. In total, 7 tumor samples were used in these analyses (*n* = 3, HSJD-GBM-001 and *n* = 4, U87).

Treatment with etomoxir significantly decreased Ki-67 and increased CPT1 expression in HSJD-GBM-001 tumors (*p* < 0.05, *p* = 0.0001, 2-way ANOVA, respectively), and decreased SLC22A5 expression in U87 tumors (*p* < 0.05, 2-way ANOVA), Figure 5c.

## 4. Discussion

Reprogrammed cellular metabolism is one of the hallmarks of cancer, including increased glucose metabolism, which has been exploited for [^18^F]FDG PET imaging in oncology. Several studies have demonstrated that fatty acid oxidation is highly active in a number of cancers, including glioma [7,8,9], and thus, similarly to glucose metabolism, imaging fatty acid metabolism might be more selective for the detection of proliferating cells. This is especially important in the management of patients with glioma, where accurate differentiation between tumor and normal brain tissue could be challenging with standard imaging modalities. To this end, we have developed a novel PET radiotracer, [^18^F]FPIA, for imaging the transcellular flux of short-chain fatty acids into tumor cells. We demonstrate that [^18^F]FPIA PET is a high-contrast approach for imaging glioma in mice by showing (a) an association of uptake with growth in multiple orthotopic glioma models, and (b) higher tumor-to-normal brain contrast compared with [^18^F]FDG. [^18^F]FPIA tumor uptake correlated with proliferation, which further supports the use of this radiotracer in monitoring tumor growth, and the expression of several lipid metabolism-associated proteins, suggesting that transcellular flux of short-chain fatty acids likely mediates the uptake of the radiotracer.

Longitudinal PET imaging of mice bearing orthotopic patient-derived HSJD-GBM-001 xenografts demonstrated a significant gradual increase in [^18^F]FPIA tumor uptake, reflecting tumor growth over time. These findings were also confirmed in two cell line-derived glioma models, U87 and LN229. The faster growing U87 tumors showed increased focal tumor uptake of [^18^F]FPIA earlier than LN229, reflecting the differences in tumor growth rates, which was also supported by bioluminescent imaging. Compared with [^18^F]FDG, [^18^F]FPIA tumor-to-normal brain contrast was 2-fold higher in the orthotopic U87 model. As anticipated, similar [^18^F]FDG uptake was observed in normal brain and tumor lesions, indicating similar glucose utilization rates and re-emphasizing the unreliability of [^18^F]FDG PET for the detection of gliomas; indeed, disease recurrence is not detected in about 40% of patients with [^18^F]FDG PET [12,13,27].

Having demonstrated suitability of [^18^F]FPIA for imaging brain tumor lesions, we examined whether tumor proliferation and the expression of lipid metabolism and transport proteins (CPT1, SLC22A2, SLC22A5, and SLC25A20), along with etomoxir treatment could relate to radiotracer uptake in HSJD-GBM-001 and U87 tumors. We selected these lipid metabolism-associated proteins based on their role in carnitine transport, which is crucial for fatty acid oxidation, and also in the elimination of pivalic acid. We found that [^18^F]FPIA tumor uptake correlated positively with proliferation indices, thus supporting the potential use of the radiotracer for imaging tumor growth. Complexity in radiotracer uptake was suggested by the positive correlation with the organic cation transporter 2 (SLC22A5) and carnitine-acylcarnitine translocase (SLC25A20), and negative correlation with the polyspecific organic cation transporter 2 (SLC22A2) and carnitine palmitoyl transferase 1 (CPT1).

SLC22A5 functions as both an organic cation transporter and a sodium-dependent high-affinity carnitine transporter, whereas SLC25A20 co-transports carnitine from the mitochondrial matrix to the intermembrane space and acyl–carnitine into the matrix; thus, our results suggest that these proteins are likely associated with radiotracer retention. In addition to its crucial role in fatty acid transport, carnitine is also involved in the detoxification and elimination of various compounds, including pivalic acid, which is mainly eliminated as pivaloyl-carnitine [19,20]. The formation of pivaloyl-carnitine is the result of pivalate activation to pivaloyl-CoA, with subsequent transfer of the pivaloyl moiety to carnitine, catalyzed by one or more of carnitine acyl transferases [20,28,29]. In normal tissues, the intracellular acyl-CoA buffering function of carnitine is accompanied by export of pivaloyl-carnitine from tissues with subsequent renal elimination [20]. Interestingly, SLC22A5 has also been implicated in pivaloyl-carnitine transport [30]; therefore, it is probable that the pivaloyl-carnitine metabolite of [^18^F]FPIA is transported from the systemic circulation into tumor cells via SLC22A5. Interestingly, SLC22A5 has been recently reported to be critical for the survival of glioma cells [31,32], supporting increased fatty acid metabolism, which is possibly mirrored in our positive correlation with [^18^F]FPIA uptake.

SLC22A2 is critical for the elimination of many endogenous cations, a wide range of drugs and environmental toxins [30,33]; therefore, it could be involved in [^18^F]FPIA elimination, leading to decreased retention, which is possibly reflected in the negative correlation with radiotracer uptake.

CPT1, which catalyzes the acyl group transfer of long-chain fatty acyl-CoA to carnitine, is overexpressed in several tumor types, and has recently been implicated to play a critical role in cancer proliferation and resistance to treatment [34]. Indeed, our results demonstrate considerable CPT1 expression in the examined tumor models, therefore, the negative correlation between CPT1 and [^18^F]FPIA tumor uptake were unexpected. Our findings could be related to cellular adaptation and differential emphasis on long- versus short-chain fatty acid metabolism and transport, which warrants further investigation. Of note, incubation of cells with [^18^F]FPIA resulted in differences in regulation of short-, medium-, and long-chain fatty acids and their carnitine derivatives [18]. Other members of the acyl transferase family, including carnitine palmitoyl transferase 2 (CPT2), carnitine octanoyl transferase (CROT), and carnitine acetyl transferase (CRAT), prefer specific chain length esters, catalyzing the reversible conversion of acyl-CoAs into acylcarnitine esters; for example, CRAT and CROT prefer short- and medium-chain acyl-CoA esters, respectively, whereas CPT1 and CPT2 prefer medium- to long-chain [34].

Furthermore, we found that acute disruption of fatty acid oxidation using the CPT1 inhibitor, etomoxir, significantly decreased [^18^F]FPIA uptake, increased CPT1 expression and decreased proliferation indices in HSJD-GBM-001 tumors. These findings further support our correlation analyses, showing that tumors with the lowest CPT1 had the highest [^18^F]FPIA uptake, suggesting that CPT1 or downstream mitochondrial effects of its inhibition by etomoxir could be directly or indirectly involved in the uptake of [^18^F]FPIA. Equally, the increase in CPT1 expression in response to a single-administration of etomoxir could be a compensatory mechanism for the irreversible inhibition of enzymatic activity. Indeed, others have also observed increased CPT1 expression, 24 h after etomoxir treatment in prostate cancer models [25]. Both PET and ex vivo biodistribution analyses showed that single-dose etomoxir caused a significant, but only minimal effect on [^18^F]FPIA uptake and proliferation, which was also reflected in some changes of expression of the lipid metabolism/transport proteins. Therefore, further studies using higher dosage or multiple-administration regimens could potentially show more drastic effects on [^18^F]FPIA uptake. While our findings showed correlations between radiotracer uptake and the expression of lipid metabolism-associated proteins, additional investigations are required to confirm whether the expression and activity of these proteins could directly influence radiotracer uptake. Interestingly, the HSJD-GBM-001 patient-derived tumors were obtained from a pediatric patient, therefore, [^18^F]FPIA could also potentially be used in the detection of pediatric tumors.

Overall, our findings support the use of [^18^F]FPIA PET for glioma imaging; however, it is important to position this information against existing technologies, including O-(2-[^18^F]-fluoroethyl)-L-tyrosine ([^18^F]FET) PET, hyperpolarized MRI, and MRS, which reflect transcellular amino acid transport, [1-^13^C]pyruvate intracellular conversion to [1-^13^C]lactate/[^13^C]bicarbonate, and the intensities of intracellular metabolites (including 2-hydroxyglutarate, N-acetyl-aspartate, choline, creatine, and phosphocreatine resonances), respectively. While [^18^F]FET appears to be valuable for the diagnostic evaluation of brain tumors, non-specific [^18^F]FET uptake in normal brain tissue has been reported in clinical studies, with passive radiotracer influx through a disrupted blood–brain barrier [35,36]. Our findings show limited uptake of [^18^F]FPIA into healthy brain of mice [18] and humans [37], which supports the notion that the radiotracer crosses the blood–brain barrier; furthermore, its use in discriminating low- versus high-grade glioma in patients is currently being evaluated (clinicaltrials.gov/ct2/show/NCT04097535, accessed on 10 May 2021). These studies, along with others on treatment response will provide insight on whether a disrupted blood–brain barrier can influence the uptake of [^18^F]FPIA. Compared with other imaging modalities, such as MRS and hyperpolarized MR, PET provides additional insight into the biology of gliomas, which can further assist in grading, delineation of tumor extent, treatment planning and monitoring. While rapidly enlarging or enhancing lesions on MRI are generally considered as tumor progression, imaging the extent of contrast enhancement has limited accuracy due to the difficulty in distinguishing tumor progression from treatment-induced changes such as necrosis [5,6]. Our studies, along with recent findings on the pivotal role of short-chain fatty acids, in particular acetate, as a preferred metabolic phenotype for energy production and proliferation in gliomas [12], support the development of [^18^F]FPIA for the detection and monitoring of glioma and potentially, other tumor types. Further studies are required to evaluate the utility of the radiotracer in the assessment of response to therapy.

## 5. Conclusions

In summary, we demonstrate a gradual increase in [^18^F]FPIA uptake in orthotopic glioma lesions over time, reflecting tumor growth, with high tumor-to-normal-brain contrast. We show that [^18^F]FPIA uptake correlates with proliferation and can be influenced by the expression of lipid metabolism and transport proteins, which requires further investigation. While single-dose etomoxir significantly decreased tumor proliferation and [^18^F]FPIA uptake, the changes were relatively minimal; therefore, additional studies are required to determine whether more drastic disruptions in fatty acid metabolism and proliferation can affect radiotracer uptake. In conclusion, [^18^F]FPIA PET could potentially assist in the diagnosis and monitoring of glioma, which can have life-changing consequences for patients.

## Figures and Tables

**Table 1:** Summary of [^18^F]FPIA tumor uptake correlations with proliferation and lipid metabolism-associated proteins.

Marker ^1^	Correlation ^2^	R^2^; *p*-Value ^3^
Ki-67	+	0.5; <0.01
CPT1	-	0.5; <0.01
SLC22A2	-	0.5; 0.005
SLC22A5	+	0.8; <0.001
SLC25A20	+	0.6; 0.001

^1^ Tumor proliferation (Ki-67) and lipid metabolism-associated protein expression (CPT1, SLC22A2, SLC22A5, SLS25A20) were assessed using immunohistochemistry. ^2^ Significant correlations between [^18^F]FPIA tumor uptake and marker expression shown as (+), positive and (-), negative. ^3^ Pearson linear regression analyses.

## Data Availability

The data presented in this study are available on request from the corresponding authors.

## References

[1] Dolecek T.A., Propp J.M., Stroup N.E., Kruchko C. (2012). CBTRUS statistical report: Primary brain and central nervous system tumors diagnosed in the United States in 2005-2009. Neuro-Oncology.

[2] Omuro A., De Angelis L.M. (2013). Glioblastoma and Other Malignant Gliomas: A Clinical ReviewGlioblastoma and Other Malignant GliomasGlioblastoma and Other Malignant Gliomas. JAMA.

[3] Ostrom Q.T., Gittleman H., Barnholtz-Sloan J.S., Wolinsky Y., Kruchko C., Xu J., Kromer C. (2016). CBTRUS Statistical Report: Primary Brain and Other Central Nervous System Tumors Diagnosed in the United States in 2009–2013. Neuro. Oncol..

[4] Jansen N.L., Graute V., Armbruster L., Suchorska B., Lutz J., Eigenbrod S., Cumming P., Bartenstein P., Tonn J.-C., Kreth F.W. (2012). MRI-suspected low-grade glioma: Is there a need to perform dynamic FET PET?. Eur. J. Nucl. Med. Mol. Imaging.

[5] Hygino da Cruz L.C., Rodriguez I., Domingues R.C., Gasparetto E.L., Sorensen A.G. (2011). Pseudoprogression and Pseudoresponse: Imaging Challenges in the Assessment of Posttreatment Glioma. Am. J. Neuroradiol..

[6] Booth T.C., Larkin T.J., Yuan Y., Kettunen M.I., Dawson S.N., Scoffings D., Canuto H.C., Vowler S.L., Kirschenlohr H., Hobson M.P. (2017). Analysis of heterogeneity in T2-weighted MR images can differentiate pseudoprogression from progression in glioblastoma. PLoS ONE.

[7] Clark P.M., Mai W.X., Cloughesy T.F., Nathanson D.A. (2016). Emerging Approaches for Targeting Metabolic Vulnerabilities in Malignant Glioma. Curr. Neurol. Neurosci. Rep..

[8] Lin H., Patel S., Affleck V.S., Wilson I., Turnbull D.M., Joshi A.R., Maxwell R., Stoll E.A. (2017). Fatty acid oxidation is required for the respiration and proliferation of malignant glioma cells. Neuro. Oncol..

[9] Pike L.S., Smift A.L., Croteau N.J., Ferrick D.A., Wu M. (2011). Inhibition of fatty acid oxidation by etomoxir impairs NADPH production and increases reactive oxygen species resulting in ATP depletion and cell death in human glioblastoma cells. Biochim. Biophys. Acta (BBA) Bioenerg..

[10] Kambach D.M., Halim A.S., Cauer A.G., Sun Q., Tristan C.A., Celiku O., Kesarwala A.H., Shankavaram U., Batchelor E., Stommel J.M. (2017). Disabled cell density sensing leads to dysregulated cholesterol synthesis in glioblastoma. Oncotarget.

[11] Mullen P.J., Yu R., Longo J., Archer M.C., Penn L.Z. (2016). The interplay between cell signalling and the mevalonate pathway in cancer. Nat. Rev. Cancer.

[12] Mashimo T., Pichumani K., Vemireddy V., Hatanpaa K.J., Singh D.K., Sirasanagandla S., Nannepaga S., Piccirillo S.G., Kovacs Z., Foong C. (2014). Acetate is a bioenergetic substrate for human glioblastoma and brain metastases. Cell.

[13] Maher E.A., Marin-Valencia I., Bachoo R.M., Mashimo T., Raisanen J., Hatanpaa K.J., Jindal A., Jeffrey F.M., Choi C., Madden C. (2012). Metabolism of [U-13 C]glucose in human brain tumors in vivo. NMR Biomed..

[14] Yoshii Y., Furukawa T., Saga T., Fujibayashi Y. (2015). Acetate/acetyl-CoA metabolism associated with cancer fatty acid synthesis: Overview and application. Cancer Lett..

[15] Schug Z.T., Vande Voorde J., Gottlieb E. (2016). The metabolic fate of acetate in cancer. Nat. Rev. Cancer.

[16] Vāvere A.L., Kridel S.J., Wheeler F.B., Lewis J.S. (2008). 1-11C-Acetate as a PET Radiopharmaceutical for Imaging Fatty Acid Synthase Expression in Prostate Cancer. J. Nucl. Med..

[17] Pisaneschi F., Witney T.H., Iddon L., Aboagye E.O. (2013). Synthesis of [^18^F]fluoro-pivalic acid: An improved PET imaging probe for the fatty acid synthesis pathway in tumours. MedChemComm.

[18] Witney T.H., Pisaneschi F., Alam I.S., Trousil S., Kaliszczak M., Twyman F., Brickute D., Nguyen Q.-D., Schug Z., Gottlieb E. (2014). Preclinical Evaluation of 3-18F-Fluoro-2,2-Dimethylpropionic Acid as an Imaging Agent for Tumor Detection. J. Nucl. Med..

[19] Brass E.P. (2002). Pivalate-generating prodrugs and carnitine homeostasis in man. Pharm. Rev..

[20] Melegh B., Kerner J., Bieber L.L. (1987). Pivampicillin-promoted excretion of pivaloylcarnitine in humans. Biochem. Pharmacol..

[21] Workman P., Aboagye E.O., Balkwill F., Balmain A., Bruder G., Chaplin D.J., Double J.A., Everitt J., Farningham D.A., Glennie M.J. (2010). Guidelines for the welfare and use of animals in cancer research. Br. J. Cancer.

[22] Vassileva V., Moriyama E.H., De Souza R., Grant J., Allen C.J., Wilson B.C., Piquette-Miller M. (2008). Efficacy assessment of sustained intraperitoneal paclitaxel therapy in a murine model of ovarian cancer using bioluminescent imaging. Br. J. Cancer.

[23] Vassileva V., Rajkumar V., Mazzantini M., Robson M., Badar A., Sharma S., Årstad E., Hochhauser D., Lythgoe M.F., Kinghorn J. (2015). Significant Therapeutic Efficacy with Combined Radioimmunotherapy and Cetuximab in Preclinical Models of Colorectal Cancer. J. Nucl. Med..

[24] Yao C.-H., Liu G.-Y., Wang R., Moon S.H., Gross R.W., Patti G.J. (2018). Identifying off-target effects of etomoxir reveals that carnitine palmitoyltransferase I is essential for cancer cell proliferation independent of β-oxidation. PLoS Biol..

[25] Schlaepfer I.R., Glodé L.M., Hitz C.A., Pac C.T., Boyle K.E., Maroni P., Deep G., Agarwal R., Lucia S.M., Cramer S.D. (2015). Inhibition of Lipid Oxidation Increases Glucose Metabolism and Enhances 2-Deoxy-2-[(18)F]Fluoro-D-Glucose Uptake in Prostate Cancer Mouse Xenografts. Mol. Imaging Biol..

[26] Bankhead P., Loughrey M.B., Fernández J.A., Dombrowski Y., McArt D.G., Dunne P.D., McQuaid S., Gray R.T., Murray L.J., Coleman H.G. (2017). QuPath: Open source software for digital pathology image analysis. Sci. Rep..

[27] Belohlávek O., Klener J., Vymazal J., Dbalý V., Tovarys F. (2002). The diagnostics of recurrent gliomas using FDG-PET: Still questionable?. Nucl. Med. Rev. Cent. East. Eur..

[28] Ohnishi S., Okamura N., Sakamoto S., Hasegawa H., Norikura R., Kanaoka E., Takahashi K., Horie K., Sakamoto K., Baba T. (2008). Role of Na+/L-carnitine Transporter (OCTN2) in Renal Handling of Pivaloylcarnitine and Valproylcarnitine Formed during Pivalic Acid-containing Prodrugs and Valproic Acid Treatment. Drug Metab. Pharmacokinet..

[29] Todesco L., Bodmer M., Vonwil K., Häussinger D., Krähenbühl S. (2009). Interaction between pivaloylcarnitine and l-carnitine transport into L6 cells overexpressing hOCTN2. Chem. Biol. Interact..

[30] Wright S.H. (2019). Molecular and cellular physiology of organic cation transporter 2. Am. J. Physiol. Ren. Physiol..

[31] Juraszek B., Czarnecka-Herok J., Nałęcz K.A. (2020). Glioma cells survival depends both on fatty acid oxidation and on functional carnitine transport by SLC22A5. J. Neurochem..

[32] Fink M.A., Paland H., Herzog S., Grube M., Vogelgesang S., Weitmann K., Bialke A., Hoffmann W., Rauch B.H., Schroeder H.W.S. (2019). L-Carnitine-Mediated Tumor Cell Protection and Poor Patient Survival Associated with OCTN2 Overexpression in Glioblastoma Multiforme. Clin. Cancer Res..

[33] Nigam S.K. (2018). The SLC22 Transporter Family: A Paradigm for the Impact of Drug Transporters on Metabolic Pathways, Signaling, and Disease. Annu. Rev. Pharm. Toxicol..

[34] Schlaepfer I.R., Joshi M. (2020). CPT1A-mediated Fat Oxidation, Mechanisms, and Therapeutic Potential. Endocrinology.

[35] Floeth F.W., Pauleit D., Sabel M., Reifenberger G., Stoffels G., Stummer W., Rommel F., Hamacher K., Langen K.J. (2006). 18F-FET PET differentiation of ring-enhancing brain lesions. J. Nucl. Med..

[36] Hutterer M., Nowosielski M., Putzer D., Jansen N.L., Seiz M., Schocke M., McCoy M., Göbel G., la Fougère C., Virgolini I.J. (2013). [^18^F]-fluoro-ethyl-L-tyrosine PET: A valuable diagnostic tool in neuro-oncology, but not all that glitters is glioma. Neuro. Oncol..

[37] Dubash S.R., Keat N., Kozlowski K., Barnes C., Allott L., Brickute D., Hill S., Huiban M., Barwick T.D., Kenny L. (2020). Clinical translation of (18)F-fluoropivalate—A PET tracer for imaging short-chain fatty acid metabolism: Safety, biodistribution, and dosimetry in fed and fasted healthy volunteers. Eur. J. Nucl. Med. Mol. Imaging.

